# Placenta Prævia

**Published:** 1902-09-20

**Authors:** 


### PLACENTA PRÆVIA.

Dr. Franklin A. Dorman[1] gives us a summary of cases of placenta prævia founded upon the statistics of the Sloane Maternity Hospital covering 11,200 deliveries. In this series there were 84 instances of placenta prævia, or one in 133⅓ cases; but, as he remarks, this ratio merely shows the high proportion of operative cases in an emergency obstetrical hospital. Multiparæ were to primiparæ as 8 to 1. Fifty-two per cent. of the patients were four gravida or over, and of the multiparæ 35 gave a history of difficult instrumental labours previously. One half of the patients were over 30 years old and seven were over 40. The bleeding at the onset was moderate and gradually increasing and independent of labour pains in 50 per cent.; in 10 per cent. it began moderately after the beginning of labour; in 40 per cent. there was sudden profuse flooding, although in one-third of these cases slight preceding attacks of bleeding had given warning signs. Of the 10 deaths four of the women were moribund on admission and died the same day shortly after delivery from loss of blood and shock. In three others death was caused by a rupture of the lower uterine segment, producing shock and hæmorrhage. One case was complicated with eclampsia. Two died of sepsis—one after rupture of the uterus. From the statistics it is concluded that the chances are so great against the child that any eagerness to save it should not lead us to adopt any treatment likely to add to the risk to the mother's life. The four cases of uterine rupture were accomplished in this way, and by men who were experts in their work, and knew the danger they were facing. As some operators now show a mortality of practically nil in Cæsarian section the applicability of this operation comes into question, and in regard to this Dr. Dorman says, "In those rare cases in which the placenta is central or nearly so, and everything in the surroundings is favourable for laparotomy, I believe the operation would be justifiable. This combination and condition, however, is very exceptionally formed."

[1] New York Medical Record, Aug. 30.

